# Validation of the parent version of the Strengths and Difficulties Questionnaire (SDQ) to screen mental health problems among school-age children in Mongolia

**DOI:** 10.1186/s12888-021-03218-x

**Published:** 2021-04-29

**Authors:** Ai Aoki, Togoobaatar Ganchimeg, Nyam Naranbaatar, Zuunnast Khishigsuren, Lkagvasuren Gundegmaa, Shagdar Bat-Erdene, Bolorchimeg Munkhbaatar, Rintaro Mori, Akihito Kikuchi, Hideaki Soya, Kiyoto Kasai, Kenji Takehara

**Affiliations:** 1 Department of Neuropsychiatry, Graduate School of Medicine, The University of Tokyo, 7-3-1 Hongo, Tokyo, Bunkyo 113-8655 Japan; 2 Department of Health Policy, National Center for Child Health and Development, 2-10-1, Okura, Tokyo, Setagaya 157-8535 Japan; 3 Department of Global Health Nursing, Faculty of Medicine, University of Tsukuba, 1-1-1, Tennodai, Tsukuba, Ibaraki 305-8577 Japan; 4 School of Nursing, Mongolian National University of Medical Sciences, Ard Ayush street, Ulaanbaatar -26. P.O.Box – 188, Ulaanbaatar, Mongolia; 5 Department of Mental Health, School of Medicine, Mongolian National University of Medical Sciences, S.Zorig street, P.O.Box – 48/11, Ulaanbaatar, 14210 Mongolia; 6 Mongolian National Institute of Physical Education, P.O. Box-224, Ulaanbaatar-13, Mongolia; 7 Graduate School of Comprehensive Human Sciences, University of Tsukuba, 1-1-1Tennodai, Tsukuba, Ibaraki 305-8577 Japan; 8 Graduate School of Medicine, Kyoto University, Yoshida-Konoecho, Sakyoku, Kyoto, Kyoto, 606-8303 Japan; 9 Sports Neuroscience Division, ARIHHP, Faculty of Health and Sport Sciences, University of Tsukuba, 1-1-1, Tennodai, Tsukuba, Ibaraki 305-8577 Japan; 10 Laboratory of Exercise Biochemistry and Neuroendocrinology, Faculty of Health and Sport Sciences, University of Tsukuba, 1-1-1, Tennodai, Tsukuba, Ibaraki 305-8577 Japan

**Keywords:** Child, Adolescent, Mental health, Low- and middle- income country, The strengths and difficulties questionnaire, Screening, Mongolia

## Abstract

**Background:**

Child and adolescent mental health problems are urgent health issues in low- and middle-income countries. To promote child and adolescent mental health services, simple validated screening tools are helpful. In Mongolia, the Strengths and Difficulties Questionnaire (SDQ), an internationally used child and adolescent mental health screening tool for children aged 4–17, was translated but not yet validated. To use the questionnaire appropriately, validation is necessary.

**Methods:**

Children at 4th year at elementary school (community sample) and children visited psychiatric outpatient service (clinical sample) were recruited and their parental version of the SDQ was compared. The discriminating ability of the parental version of the SDQ was examined using Receiver Operating Characteristics (ROC) analysis on the SDQ total difficulties score. The area under the ROC curve (AUC) was used as a measure. Cut-off score was determined by normative banding that categorizes children with the highest 10% score range as abnormal and the second highest 10% as borderline following the original method; this cut-off score was compared with the cut-off score candidates with good balance between sensitivity and specificity using ROC analysis.

**Results:**

We included 2301 children in the community sample, and 429 children in the clinical sample. Mean age was 9.7 years (SD 0.4, range 8.3–12.0) among the community sample and 10.4 years (SD 3.8, range 4.0–17.8) among the clinical sample. The mean total difficulties score was 12.9 (SD 4.8) among the community sample and 20.4 (SD 6.2) among the clinical sample. A total of 88.8% of the community sample and 98.8% of the clinical sample answered the SDQ. Using ROC analysis, the AUC was 0.82 (95% confident interval 0.80–0.85), which meant moderate discriminating ability. Using normative banding, the borderline cut-off score was 16/17 and abnormal cut-off score was 19/20. For cut-off scores of 16/17 and 19/20, sensitivity was 71.9 and 53.8% and specificity was 78.5 and 90.5%, respectively. The cut-off score candidates by ROC analysis were 16/17 and 17/18.

**Conclusions:**

The parental version of the SDQ had moderate discriminating ability among Mongolian school-age children. For the screening of mental health problems among community children, cut-off score of 16/17 is recommended.

**Supplementary Information:**

The online version contains supplementary material available at 10.1186/s12888-021-03218-x.

## Background

Children and adolescents comprise a third of the world’s population, and 10–20% of them are considered to suffer from mental health problems [1, 2]. According to the Global Burden of Disease study, mental disorders and substance use disorders account for 15–30% of years lived with disability among young people [3]. Despite the growing burden of disease and its long-lasting consequences beyond childhood and adolescence, there are significant gaps between need and resource availability, particularly in low- and middle-income countries (LMICs) where 90% of the world’s children and adolescents live [1]. Studies of mental health services suggest that a small proportion of those with mental health needs are receiving care [4–6].

Valid and simple screening instruments can contribute to the promotion of child and adolescent mental health services in LMICs in multiple ways. For example, it enables an identification of people with service needs at health care facilities as well as an epidemiological surveillance at the population level.

The Strengths and Difficulties Questionnaire (SDQ) is a mental health screening instrument for children and adolescents aged 4–17 years old used in over 100 countries including LMICs [7–15]. The SDQ is extensively used in both research and clinical settings as it is quick and easy to complete and score has good psychometric properties [16]. For the screening of psychiatric disorders, the SDQ has been shown to have 39–72% sensitivity and 76–94% specificity by the borderline/abnormal cut-off score in other languages [16–18]. Moreover, it has very broad acceptance by non-health professionals, children and their parents [19]. However, normative scoring and psychometric properties of the SDQ have been extensively assessed predominantly in samples from high-income countries. Several cross-cultural issues have been demonstrated. In different cultural contexts, the same answer may be perceived different ways, mental health terminologies may not be translated well, the original cut-off scores do not work appropriately, and the original five-factor structure may not be replicated [14, 18, 20–23].

Mongolia is a lower middle-income country. The epidemiological transition from communicable diseases to non-communicable diseases is occurring and mental health needs are assumed to be high [24]. The Mongolian version of the SDQ was developed through translation and back-translation and made available on the official website [25]. The SDQ is by far the only one internationally used mental health screening instrument available in Mongolia. However, validation of the Mongolian version of the SDQ has not been conducted yet, and the international cut-off scores, which were originally the cut-off scores derived from the UK children, have been used. A previous study in Mongolia demonstrated that 43% of adolescents were classified as abnormal by the international cut-off score [26]. There is a strong need for validating the SDQ and its cut-off score in Mongolia.

Therefore, the present study aimed to analyze the discriminative validity of the parent version of the Mongolian SDQ and to define the appropriate cut-off scores for the categories in Mongolia by banding normative data. Previous studies validated the SDQ by various methods such as comparing the results of the SDQ to other gold standard questionnaires, to psychiatric diagnosis given by established structured diagnostic interviews, comparing the results of the SDQ between low- and high-risk population. The present study aimed to validate the SDQ by comparing low- and high-risk population despite the risk of contamination of children with and without mental health problems, as there is no other gold standard screening tools or structured diagnostic interviews available in Mongolia. Appropriate cut-off scores and proven validity of the SDQ are necessary for the effective use of the SDQ in Mongolia for various purposes such as epidemiological surveillance and clinical needs assessment.

## Methods

### Study settings

This study compared two samples to validate the SDQ. These are: (1) a community sample; and (2) a clinical sample. The community sample consisted of children recruited from public schools in one district in the capital city. The clinical sample consisted of children recruited at a psychiatric outpatient service at the National Mental Health Center in Mongolia, which is the only one specialized service for child and adolescent psychiatry in Mongolia. There was no gold standard questionnaire to compare with the SDQ in Mongolia. In addition, there were no known groups which consist only of children with mental disorders and only of children without mental disorder. Thus, in the present study, we aimed to validate the SDQ through examining the discriminating ability of the SDQ between a community sample and clinical sample although there was a possibility that the community sample might include children with mental health problems and the clinical sample might include children without mental health problem. This method for SDQ validity has been applied in previous studies [27, 28].

### Community sample

The community sample consisted of participants in a study that evaluated the effectiveness of physical activity on academic achievement and cognitive function among children in elementary schools. The details of this study are described elsewhere [29]. Participants were children in their 4th year at 10 public primary schools in Sukhbaatar district, which is one of nine districts in the capital city, Ulaanbaatar. Inclusion criteria for the original study were: (1) attendance at a public school in the Sukhbaatar district; (2) written consent from parents or guardians; and (3) child’s age-appropriate literacy in Mongolian. Exclusion criteria were: (1) comorbidities or contraindications prohibiting participation in an exercise program; and (2) enrollment in a special needs program. The population of the district is roughly 10 and 5% of the population of Ulaanbaatar and Mongolia, respectively [30]. The district stretches from urban city center to non-urban area where the infrastructure is not enough developed so that the socioeconomical background of the participants are diverse. There is no apparent difference in terms of population structure and residents’ economic level in this district compared with other districts in Ulaanbaatar [30].

### Clinical sample

The clinical sample was recruited at the child and adolescent mental health outpatient service at the National Mental Health Center. The National Mental Health Center is a tertiary-level hospital and it is the only one specialized hospital for mental health in Mongolia. Almost all children with severe mental disorders or intellectual disability in Ulaanbaatar are considered to visit this National Mental Health Center.

The inclusion criteria were: (1) younger than 20 years old; (2) visiting Child and Adolescent Mental Health Outpatient Service at the National Mental Health Center between 1st December 2018 to 31st March 2019; (3) written consent from parents or guardians; and (4) parents’ or guardians’ literacy in Mongolian. There were no exclusion criteria. Originally, this sample was recruited to validate the SDQ as well as to understand the global characteristics of the users, all the target age range (younger than 20 years old) were recruited. However, in this analysis, children aged between 4 to 17 years old were included in the analysis due to the target age range of the SDQ.

### Measures

Socio-demographic characteristics and the SDQ were obtained from a parent or guardian in both community and clinical samples. Socio-demographic characteristics included age, sex, region, maternal education, family structure, and household income. Clinical diagnosis was obtained in the clinical sample.

### The strengths and difficulties questionnaire

The SDQ is a 25-item questionnaire for child and adolescent mental health problems. It is for 4–17-year-old children and adolescents. It is used for clinical assessment, epidemiological study and screening of psychiatric disorders. The 25-items are answered using a 3-point scale, “certainly true”, “somewhat true” and “not true” and scored from 0 to 2 points. The items yield 5 subscale scores that range from 0 to 10 including: (1) emotional symptoms; (2) conduct symptoms; (3) hyperactivity/inattention; (4) peer relationship problems; and (5) prosocial behavior. Summing emotional, conduct, hyperactivity/inattention and peer relationship subscale scores yields a total difficulties score that ranges from 0 to 40. The SDQ uses cut-off scores that are defined using normative data banding in three categories: normal (80th percentile and less), borderline (80th-90th percentile) or abnormal (90th percentile and more). The Mongolian version was obtained from the official website [25].

In the clinical sample, clinical diagnosis was made by certified psychiatrists at the hospital according to the 10th revision of International Statistical Classification of Diseases and Related Health Problems (ICD-10). The diagnosis was obtained by attending consultation or reviewing medical records. The ICD-10 is used conventionally in Mongolia and was used in the present study.

### Statistical analysis

Descriptive analysis was done for socio-demographic characteristics. Two samples were compared of its socio-demographic background. For age, t-test was done. For other categorical variables, chi-square test was done.

### Factor structure and internal consistency analysis

Among the community sample, exploratory factor analysis (EFA) was performed. To decide the number of factors, Minimum Average Partial (MAP) criterion, Bayesian Information Criterion (BIC), and the number of components by parallel analysis was used. When there were multiple candidates of factor number, the factor number was determined assessing factor loadings of each models.

Using the factor number determined by the exploratory factor analysis, McDonald’s omega coefficient was calculated for the entire SDQ and each subscale. Omega values of 0.7–0.8 are considered sufficient and above 0.8 are considered good [31, 32].

The SDQ is consisted of five subscales. Confirmatory factor analysis (CFA) was performed among community sample to examine the original five-factor structure. To evaluate the model fit, the Comparative Fit Index (CFI), the Tucker Lewis Index (TLI) and the Root Mean-Square Error of Approximation (RMSEA) were calculated. For TLI and CFI, values lower than 0.9 is considered as lack of fit, 0.90–0.95 as reasonable fit, and 0.95–1.00 as good fit [33]. For RMSEA, values smaller than 0.05 is an indicator of good fit, 0.05–0.08 is reasonable fit [33].

The analysis was done with lavaan library and psych library on R version 3.4.4 [34, 35].

### Receiver operating characteristic analysis

To validate the SDQ, Receiver Operating Characteristic (ROC) analysis was performed. ROC curves are curves drawn by plotting sensitivity and specificity for all possible thresholds. The discriminating ability of the total difficulties score between the community and clinical samples was assessed by evaluating the area under the curve (AUC). The AUC of total difficulties scores was calculated among the entire sample, subdividing by sex. AUC values of 1.0 means perfect discriminating ability and AUC values of 0.5 means no discriminating ability at all. Conventionally, AUC values of 0.5–0.7 are considered low accuracy, 0.7–0.9 are considered moderate accuracy and 0.9–1.0 are high accuracy [36]. The analysis was done with pROC library on R version 3.4.4 [37].

This analysis had an assumption that the prevalence of psychiatric disorders among the clinical sample was substantially higher than that of the community sample. This analysis did not have an assumption that either none of the participants in the community sample had a psychiatric disorder or all the participants in the clinical sample had a psychiatric disorder.

To assess the discriminating ability of subscale scores, AUC of each subscale score was calculated.

Although the clinical sample consisted of patients at a child and adolescent psychiatric outpatient service, some participants in the clinical sample might not have a psychiatric disorder. If many in the clinical sample did not have a psychiatric disorder, it might be difficult to examine the discriminating ability of the SDQ. To solve this problem, a sensitivity analysis was conducted using the community sample and a subsample of the clinical sample participants which only included those with definite psychiatric diagnoses.

### Cut-off score by normative banding

Normative data for the SDQ total difficulties score of the entire community sample were described. As the etiology of child and adolescent mental health problems has sex differences, normative data by sex were also described (Supplementary Table 1) [38]. Normative data of the 5 subscale scores were described (Supplementary Table 2).

To determine the original UK version SDQ cut-off scores, banding of the normative data of the SDQ total difficulties scores was done to divide percentiles into abnormal and borderline categories [28]. In the present study, the same banding method was applied to the normative data to determine the cut-off scores of the Mongolian version.

### Comparison with the cut-off score candidates by ROC analysis

The cut-off score by normative banding was compared with the cut-off score candidates using ROC analysis which has a balance between sensitivity and specificity. For ROC analysis, the best cut-off score was analyzed by two methods: (1) determining the point closest to the top-left point of the plot which means perfect discriminating ability (100% sensitivity and 100% specificity); and (2) Youden’s J statistics which uses the point that maximizes the distance to the line of no discriminating ability (connecting the point of 100% sensitivity and 0% specificity and the point of 0% sensitivity and 100% specificity) [36, 39, 40]. The candidates from the ROC determined cut-off score were compared with the cut-off score by normative banding.

### Sensitivity and specificity

Though we did not have an assumption that either the community sample did not include any participants with psychiatric disorders or that the clinical sample did not include any participants without psychiatric disorders, sensitivity, specificity, positive likelihood ratio, and negative likelihood ratio to discriminate participant’s group (clinical sample or community sample) were calculated for each cut-off score. Sensitivity meant the proportion of above threshold participants in the clinical sample. Specificity meant the proportion of below threshold children in the community sample The proportion of children above each cut-off score was calculated.

## Results

### Community sample

A total of 2309 children were enrolled in met the inclusion criteria. None of the 2309 children met exclusion criteria. Of 2309 children, 2301 children participated in the study on physical activity, academic achievement and cognitive function (99.6%). A total of eight children did not participate because their parents/guardians did not provide an informed consent. Data from 2301 children were analyzed in this analysis.

### Clinical sample

**Fig. 1:**
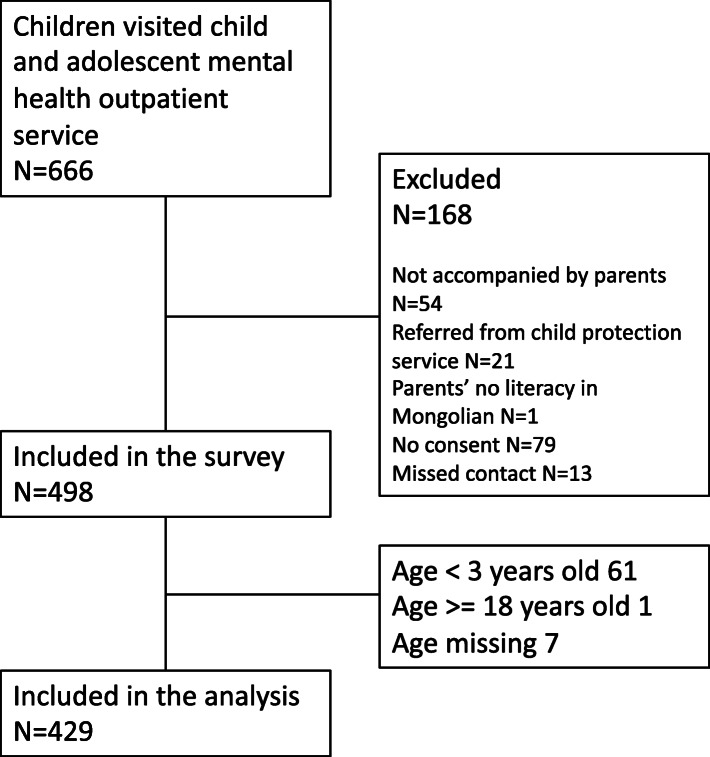
Participants in the clinical sample

During the study period 666 children visited the National Mental Health Center, and 498 participated in the study (74.8%). Of those, 429 participants were between 4 and 17 years and were included in this analysis. Participant’s flow is presented in Fig. 1.

### Age, sex and socioeconomic factors

The mean age of the community sample was 9.7 years (SD 0.4, range 8.3–12.0). Of all participants, 51.3% were male. All community sample participants were living in Ulaanbaatar, 80.3% were living in a two-parent family, 94.0% had a mother whose educational level was upper secondary school or more, and 65.1% had household income above 700,000 MNT. Mean age of the clinical sample was 10.4 years (SD 3.8, range 4.0–17.8) and 60.1% were male. Of all clinical sample participants, 84.5% were living in Ulaanbaatar, 71.0% were living in a two-parent family, 88.2% had a mother whose educational level was upper secondary school or more, and 64.9% had household income above 700,000 MNT.

Mean age was higher in the clinical sample (*p* < 0.001) and the proportion of categories of sex, region, family structure, maternal education differed between two samples (*p* = 0.001, *p* < 0.001, *p* < 0.001, *p* < 0.001 respectively). The proportion of categories of household income did not differ between two samples (*p* = 1.00). Participants’ socio-demographic characteristics are presented in Table 1.

**Table 1:** Socio-demographic characteristics of participants

	Community sample		Clinical sample		*P* value
	*N*	%	*N*	%	
Total number	2301		429		
Sex					0.001
Male	1051	51.3	258	60.1	
Female	997	48.7	171	39.9	
Missing	253	–	0	–	
Age (Mean/SD)	9.7	0.4	10.4	3.8	< 0.001*
< 5	0	0	31	7.2	
5–9	1569	77.1	174	40.6	
10–14	467	22.9	154	35.9	
15–19	0	0	70	16.3	
Missing	265		0		
Region					< 0.001
Ulaanbaatar	2301	100.0	344	84.5	
Outside Ulaanbaatar	–		63	15.5	
Missing	0		22	–	
Family structure					< 0.001
Two-parent	1644	80.3	303	71.0	
One-parent	278	13.6	104	24.4	
No parent	126	6.2	20	4.7	
Missing	253	–	2	–	
Maternal education					< 0.001
Lower secondary and less	113	6.0	49	11.8	
Upper secondary and more	1786	94.0	367	88.2	
Missing	402	–	13	–	
Household income					1.00
700,000 MNT and less^**^	708	34.9	149	35.1	
700,001 MNT and more^**^	1319	65.1	276	64.9	
Missing	274	–	4	–	

* t-test was conducted for age of two samples

**MNT Mongolian Tugrik. 700,000 MNT was about 280 USD at the exchange rate in 2017–2019

### Clinical diagnosis

The psychiatric diagnoses of the children in the clinical sample were 123 (28.7%) with F7: Mental retardation, 74 children (21.0%) with F8: Disorders of psychological development and 66 (18.7%) with F9: Behavioral and emotional disorders with onset usually occurring in childhood and adolescence and 76 children (17.7%) were missing data which included no psychiatric diagnosis, not having determined a diagnosis or had missing values.

### Discrimination between the community and clinical sample

Among the community sample, 2046 participants (88.9%) answered the SDQ. The mean of the total difficulties score of the community sample was 12.9 (SD 4.8). Among the clinical sample, 424 participants (98.8%) answered the SDQ. The mean of the total difficulties score of the clinical sample was 20.4 (SD 6.2).

### Factor structure and internal consistency

The number of factors was two by MAP, five by BIC, and four by parallel analysis. When the factor loadings of each model were examined, four-factor model is in line with the original structure better, and 33% of variance was explained. In the four-factor model, emotion and peer problem subscales ware not distinguished. Internal consistency was sufficient for the entire SDQ but poor for each subscale by omega coefficient (the entire SDQ 0.75, conduct subscale 0.48, hyper activity subscale 0.65, emotional subscale 0.53, peer relationship subscale 0.37, and prosocial subscale 0.41).

CFA was conducted among community sample. CFA demonstrated a misfit when evaluated by CFI and TLI (CFI = 0.73, TLI = 0.69), but reasonable fit by RMSEA (RMSEA = 0.054 (95%CI 0.051–0.056)).

### Discrimination between the community and clinical sample

**Fig. 2:**
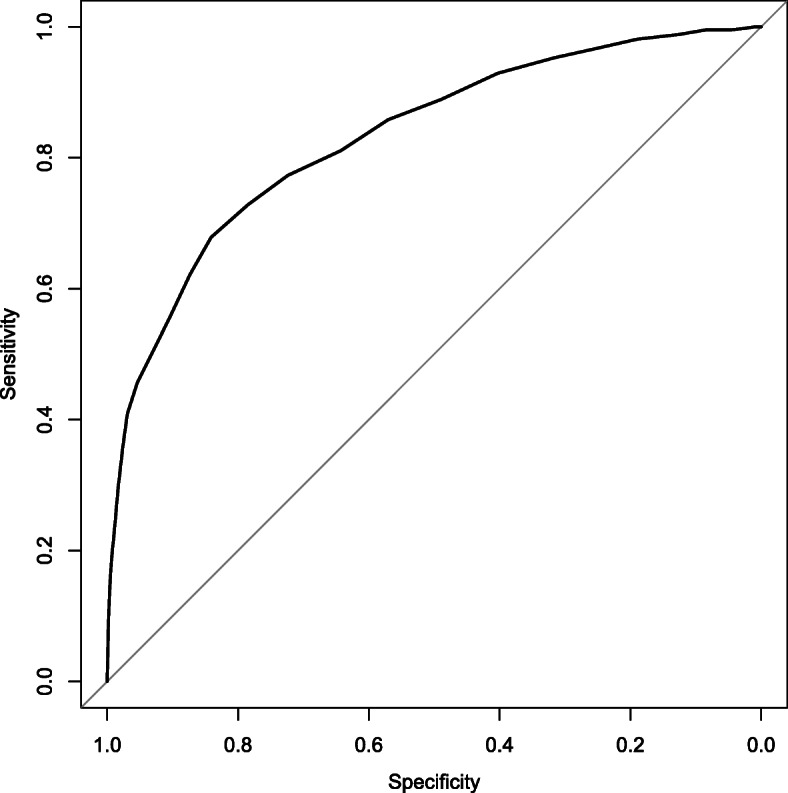
ROC curve of total difficulties score

**Fig. 3:**
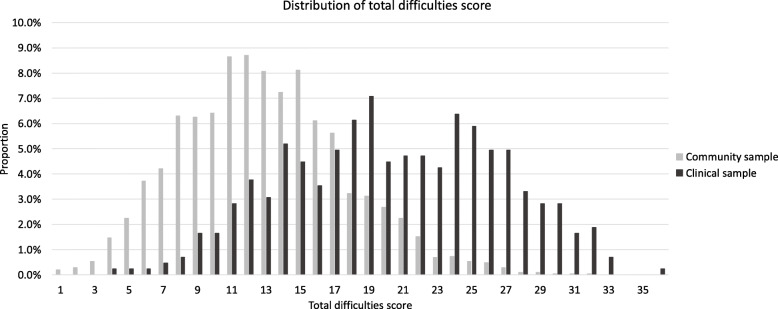
Distribution of total difficulties score

For ROC analysis, the area under the curve (AUC) was 0.82 (95% confidential interval (95% CI) 0.80–0.85), and the 95% CI was estimated by the DeLong method and 2000 stratified bootstrap replicates and the results were the same (Fig. 2). Distribution bar graph of the total difficulties score is presented in Fig. 3.

Among males, the mean total difficulties score was 13.3 (SD 4.9) in the community sample and 20.4 (SD 6.1) in the clinical sample (Supplementary Fig. 1). AUC was 0.81 (95% CI 0.78–0.84). Among females, the mean total difficulties score was 12.4 (SD 4.6) in community sample and 20.4 (SD 6.5) in the clinical sample (Supplementary Fig. 1). AUC was 0.84 (95% CI 0.80–0.87). This meant that the higher mean total difficulties score in the clinical sample was not due to the higher proportion of males in the clinical sample.

For each subscale score, AUC was calculated: (1) emotional subscale 0.67 (95% CI 0.64–0.70); (2) conduct subscale 0.76 (95% CI 0.73–0.79); (3) hyperactivity/ inattention subscale 0.74 (95% CI 0.71–0.77); (4) peer relationship subscale 0.78 (95% CI 0.76–0.81); and (5) prosocial subscale 0.67 (95% CI 0.64–0.70).

A total of 350 participants among the clinical sample had a definite diagnosis of a psychiatric disorder. Using a subsample of the clinical sample, which only included participants with definite psychiatric diagnosis, AUC of the total difficulties score was 0.82 (95%CI 0.80–0.85), which was consistent with the original AUC value. For each subscale score, AUC was calculated: (1) emotional subscale 0.68 (95% CI 0.65–0.71); (2) conduct subscale 0.74 (95% CI 0.71–0.77); (3) hyperactivity/ inattention subscale 0.72 (95% CI 0.69–0.75); (4) peer relationship subscale 0.78 (95% CI 0.75–0.80); and (5) prosocial subscale 0.67 (95% CI 0.63–0.70). These were similar to the results of the original analysis.

### Cut-off score by normative banding

Among the entire community sample, the cut-off score between normal and borderline and between borderline and abnormal was 16/17 and 19/20 respectively. Cut-off scores by normative banding were compared to that of the UK [12]. Cut-off scores of subscales are presented in Table 2. The cut-off score for the total difficulties score was 3 points higher than that of the UK. The cut-off scores of emotion, conduct and hyperactivity/ inattention and peer relationship subscales were 0–2 points higher than those of the UK. The cut-off score for the prosocial subscale was 2 points lower than that of UK.

**Table 2:** Normative banding

	Mongolia			UK*		
	Normal	Borderline	Abnormal	Normal	Borderline	Abnormal
Total difficulties score	0–16 (78.5%)	17–19 (12.0%)	20–40 (9.5%)	0–13 (82.1%)	14–16 (8.2%)	17–40 (9.8%)
Emotion subscale	0–5 (80.5%)	6 (9.8%)	7–10 (9.8%)	0–3 (80.8%)	4 (7.8%)	5–10 (12.4%)
Conduct subscale	0–2 (72.2%)	3 (15.5%)	4–10 (12.3%)	0–2 (76.4%)	3 (10.9%)	4–10 (12.7%)
Hyperactivity subscale	0–6 (81.3%)	7 (8.3%)	8–10 (10.4%)	0–5 (77.9%)	6 (7.4%)	7–10 (14.7%)
Peer relationship subscale	0–4 (83.7%)	5 (8.8%)	6–10 (7.5%)	0–2 (78.0%)	3 (10.2%)	4–10 (11.7%)
Prosocial subscale	6–10 (85.4%)	5 (8.8%)	0–4 (5.7%)	8–10 (79.5%)	7 (10.0%)	0–6 (10.6%)

*Cut-off scores from UK were obtained from the SDQ official website [12].

Normative data for the SDQ total difficulties score were demonstrated in Supplementary Table 1. Normative data of the five subscale scores were described in Supplementary Table 2.

### Comparison with cut-off score candidates by ROC analysis

For the first method, the best cut-off score was determined by the point closest to the top-left point and was 16/17. Sensitivity was 0.72 and specificity was 0.78. For the second method, the Youden method, the cut-off score was 17/18. Sensitivity was 0.67 and specificity was 0.84. According to the comparison between cut-off scores by normative banding and these cut-off score candidates using ROC analysis, the cut-off score of 16/17 was considered to have better balance between sensitivity and specificity than the cut-off score of 19/20. The cut-off score of 19/20 weighs more on specificity. Thus, the cut-off score of 16/17 is considered to be a good cut-off score for the screening of mental health problem among community children in Mongolia.

### Sensitivity and specificity

For each cut-off score, sensitivity, specificity, positive likelihood ratio, negative likelihood ratio and proportion of high risk among the community sample were calculated and displayed in Table 3.

**Table 3:** Indicators of cut-off score candidates

Indicator	Cut-off score	
	16/17	19/20
Sensitivity	71.9%	53.8%
Specificity	78.5%	90.5%
Positive likelihood ratio	3.34	5.64
Negative likelihood ratio	0.36	0.51
Proportion of high risk among community sample	21.5%	9.5%

## Discussion

### Summary of results

The SDQ score of 2301 community representative children and 429 mental health service user representative children were compared. The AUC value of the total difficulties score was 0.82, which means moderate discriminating ability. As for cut-off scores, normative banding suggested 16/17 for a cut-off between normal and borderline and 19/20 for a cut-off between borderline and abnormal. Both cut-off scores were three points higher than the international cut-off scores. The cut-off score of 16/17 had good balance between sensitivity and specificity by ROC analysis. We recommend a cut-off score of 16/17 for the screening of mental health problems among community children. This analysis demonstrated that the use of international cut-off scores in Mongolia leads to an over estimation of high risk children.

### Comparison with previous studies

Previous validation studies of the parental version of the SDQ has demonstrated AUC ranges between 0.66 to 0.87, which moderated discriminating ability [13, 17, 18, 28, 41]. AUC of the parental version of the Mongolian SDQ was 0.82 and consistent with previous studies. This suggested that the parental version of the SDQ could be used in Mongolia.

EFA suggested a four-factor structure. Four factor structure was suggested by a study in the Netherland [42]. Internal consistency was sufficient for the entire SDQ but low for subscales. A systematic review reported that some studies demonstrated a factor structure other than the original five-factor structure or a low internal consistency for some subscales [23]. CFA did not show a good fit for five-factor structure by CFI and TLI. However, some of the previous studies also failed to show five-factor structure [23]. Hence, the subscales need to be used with caution.

### Cut-off score

Internationally, normative banding has been used to determine the cut-off score of the SDQ. However, ROC analysis suggested that cut-off scores between borderline and abnormal disproportionately weighed on specificity rather than sensitivity and false negatives might be a problem. Although this study suggests a cut-off score of 16/17 for normal/borderline and 19/20 for borderline/abnormal following the methods of previous studies, other cut-off scores can also be considered according to the purpose and nature of the target population. For example, if a human resource to perform an assessment for screened children is depleted, minimizing false negative is an important strategy. In that case, higher cut-off score must be considered.

### Difference from the results of previous survey in Mongolia

One previous study has used the SDQ parental version among Mongolian adolescents [26]. In the study, children aged 11 to 18 from both Ulaanbaatar and outside Ulaanbaatar were included. The mean total difficulties score was 16.6 (SD 4.4) by parental report. This mean score was higher than the mean total difficulties score among the current community sample. The difference in age range and residential area of the study sample might explain the difference.

### Limitations

The community sample consisted of children attending the same year at primary school and did not include younger children or adolescents. The community sample consisted of children at around 9–10 years old, which is childhood. Thus, the mental health problems occurring in the adolescence is not captured. In this study, we used children living in Ulaanbaatar as the community sample. However, the lifestyle of children is very diverse in urban and rural areas. As the suggested cut-off scores are based on the normative banding of the community sample, cut-off scores among different age range samples and rural area samples are not confirmed. Confirming them will be a future research focus. In addition, as the data collection period was not year-round, there might have been seasonal effects.

In our study, the community sample might have included children with mental health problems and the clinical sample might have included children without mental health problems. Regarding the community sample, if the community sample had exclusively consisted of children without mental disorders, the discriminating ability would have been higher and we did not overestimate the discriminating ability. Similarly, for the clinical sample, it did not exclusively consist of children with mental disorders. However, the sensitivity analysis using only children with definite psychiatric disorder yielded the same level of discriminating ability. Thus, the present study did not overestimate the discriminating ability due to the sampling methods.

## Conclusions

The parental version of the SDQ demonstrated moderate discriminating ability among Mongolian school-age children. The cut-off score between normal and borderline was 16/17 and between borderline and abnormal was 19/20. For the screening of mental health problems among community children, the cut-off score 16/17 is recommended. The suggested cut-off score was considerably different from the cut-off score used internationally. If the internationally used cut-off score is used in Mongolia, specificity would be very low and false positives would be more likely. Confirmation of cut-off score for early childhood, adolescence, and rural population will be a future research focus.

## Supplementary Information

**Additional file 1:** Distribution of total difficulties score by sex.

**Additional file 2: Supplementary Table 1**. Normative data of the SDQ total difficulties score.

**Additional file 3: Supplementary Table 2**. Normative data of subscales.

## Data Availability

The datasets generated and/or analyzed during the current study are not publicly available but are available from the corresponding author on reasonable request.

## Abbreviations

AUC: Area under the curve
BIC: Bayesian Information Criterion
CFA: Confirmatory factor analysis
CFI: Comparative Fit Index
EFA: Exploratory factor analysis
ICD-10: The 10th revision of International Statistical Classification of Diseases and Related Health Problems
LMIC: Low- and middle- income country
MAP: Minimum Average Partial
RAMSEA: Root Mean-Square Error of Approximation
ROC: Receiver Operation Characteristics
SDQ: Strengths and Difficulties Questionnaire
TLI: Tucker Lewis Index
95% CI: 95% confidential interval

## References

[1] Kieling C, Baker-Henningham H, Belfer M, Conti G, Ertem I, Omigbodun O, Rohde LA, Srinath S, Ulkuer N, Rahman A (2011). Child and adolescent mental health worldwide: evidence for action. Lancet.

[2] Morris J, Belfer M, Daniels A, Flisher A, Ville L, Lora A (2011). Treated prevalence of and mental health services received by children and adolescents in 42 low-and-middle-income countries. J Child Psychol Psychiatry.

[3] Institute for Health Metrics and Evaluation (IHME). GBD Compare. Seattle: IHME, University of Washington; 2015. Available from http://vizhub.healthdata.org/gbd-compare.

[4] Patel V, Kieling C, Maulik PK, Divan G (2013). Improving access to care for children with mental disorders: a global perspective. Arch Dis Child.

[5] Paula CS, Bordin IA, Mari JJ, Velasque L, Rohde LA, Coutinho ES (2014). The mental health care gap among children and adolescents: data from an epidemiological survey from four Brazilian regions. PLoS One.

[6] Borges G, Benjet C, Medina-Mora M, Orozco R, Wang P (2008). Treatment of mental disorders for adolescents in Mexico City. Bull World Health Organ.

[7] Malhotra S, Kohli A, Kapoor M, Pradhan B (2009). Incidence of childhood psychiatric disorders in India. Indian J Psychiatry.

[8] Syed EU, Hussein SA, Mahmud S (2007). Screening for emotional and behavioural problems amongst 5-11-year-old school children in Karachi, Pakistan. Soc Psychiatr Psychiatr Epidemiol.

[9] Xiaoli Y, Chao J, Wen P, Wenming X, Fang L, Ning L, Huijuan M, Jun N, Ming L, Xiaoxia A, Chuanyou Y, Zenguo F, Lili L, Lianzheng Y, Lijuan T, Guowei P (2014). Prevalence of psychiatric disorders among children and adolescents in Northeast China. PLoS One.

[10] Mohammadi MR, Arman S, Khoshhal Dastjerdi J, Salmanian M, Ahmadi N, Ghanizadeh A, Alavi A, Malek A, Fathzadeh Gharibeh H, Moharreri F, Hebrani P, Motavallian A (2013). Psychological problems in Iranian adolescents: application of the self report form of strengths and difficulties questionnaire. Iran J Psychiatry.

[11] Menezes RC, Lira PI, Leal VS, Oliveira JS, Santana SC, Sequeira LA (2011). Determinants of stunting in children under five in Pernambuco, northeastern Brazil. Rev Saude Publica.

[12] youthinmind: SDQinfo. https://www.sdqinfo.com/ Accessed 29 Jan 2021.

[13] Salayev K, Rustamov I, Gadjiyeva N, Salayev R, Sanne B (2016). The discriminative ability of the Azeri version of the strengths and difficulties questionnaire in outpatient practice. Community Ment Health J.

[14] Hoosen N, Davids EL, de Vries PJ, Shung-King M (2018). The strengths and difficulties questionnaire (SDQ) in Africa: a scoping review of its application and validation. Child Adolesc Psychiatry Ment Health.

[15] Du Y, Kou J, Coghill D (2008). The validity, reliability and normative scores of the parent, teacher and self report versions of the strengths and difficulties questionnaire in China. Child Adolesc Psychiatry Ment Health.

[16] Goodman R (2001). Psychometric properties of the strengths and difficulties questionnaire. J Am Acad Child Adolesc Psychiatry.

[17] He JP, Burstein M, Schmitz A, Merikangas KR (2013). The strengths and difficulties questionnaire (SDQ): the factor structure and scale validation in U.S. adolescents. J Abnorm Child Psychol.

[18] Borg AM, Kaukonen P, Joukamaa M, Tamminen T (2014). Finnish norms for young children on the strengths and difficulties questionnaire. Nord J Psychiatry.

[19] Goodman R, Scott S (1999). Comparing the strengths and difficulties questionnaire and the child behavior checklist: is small beautiful?. J Abnorm Child Psychol.

[20] Kersten P, Dudley M, Nayar S, Elder H, Robertson H, Tauroa R, McPherson KM (2016). Cross-cultural acceptability and utility of the strengths and difficulties questionnaire: views of families. BMC Psychiatry.

[21] Lai KY, Luk ES, Leung PW, Wong AS, Law L, Ho K (2010). Validation of the Chinese version of the strengths and difficulties questionnaire in Hong Kong. Soc Psychiatry Psychiatr Epidemiol.

[22] Hawes DJ, Dadds MR (2004). Australian data and psychometric properties of the strengths and difficulties questionnaire. Aust N Z J Psychiatry.

[23] Stone LL, Otten R, Engels RC, Vermulst AA, Janssens JM (2010). Psychometric properties of the parent and teacher versions of the strengths and difficulties questionnaire for 4- to 12-year-olds: a review. Clin Child Fam Psychol Rev.

[24] World Health Organization. World Health Organization - Noncommunicable Diseases (NCD) Country Profiles Mongolia, 2014.

[25] youthinmind. Downloadable SDQ and related items, Mongolian. One-sided SDQ for parents or teachers of 4–17 year olds. 2020. https://www.sdqinfo.org/py/sdqinfo/b3.py?language=Mongolian. Accessed 29 Jan 2021.

[26] Bayarmaa V, Tuya N, Batzorig B, Guljanat Y, Altanzul N, Soyolmaa B, et al. Prevalence of Emotional and Behavioral Problems among Adolescence and Some Risk Factors. J Mental Disord Treat. 2017;03(01). 10.4172/2471-271X.1000136.

[27] Goodman R, Meltzer H, Bailey V (2003). The strengths and difficulties questionnaire: a pilot study on the validity of the self-report version. Int Rev Psychiatr.

[28] Goodman R (1997). The strengths and difficulties questionnaire: a research note. J Child Psychol Psychiatry.

[29] Takehara K, Ganchimeg T, Kikuchi A, Gundegmaa L, Altantsetseg L, Aoki A, Fukuie T, Suwabe K, Bat-Erdene S, Mikami M, Mori R, Soya H (2019). The effectiveness of exercise intervention for academic achievement, cognitive function, and physical health among children in Mongolia: a cluster RCT study protocol. BMC Public Health.

[30] National Statistics Office of Mongolia. Mongolian Statistical Information Service 2019. https://www.1212.mn/. Accessed 29 Jan 2021.

[31] Stone LL, Janssens JM, Vermulst AA, Van Der Maten M, Engels RC, Otten R (2015). The strengths and difficulties questionnaire: psychometric properties of the parent and teacher version in children aged 4-7. BMC Psychol.

[32] Evers A, Sijtsma K, Lucassen W, Meijer RR (2010). The Dutch review process for evaluating the quality of psychological tests: history, procedure, and results. Int J Test.

[33] Brown TA. Confirmatory Factor Analysis for Applied Research. 2nd Edition ed: Guilford Press; 2015.

[34] Rosseel Y, Jorgensen TD, Oberski D, Byrnes J, Vanbrabant L, Savalei V, et al. Package “lavaan”. 2020. https://cran.r-project.org/web/packages/lavaan/lavaan.pdf. Accessed 29 Jan 2021.

[35] Revelle W. Package “psych”. 2020. https://personality-project.org/r/psych-manual.pdf. Accessed 29 Jan 2021.

[36] Akobeng AK (2007). Understanding diagnostic tests 3: receiver operating characteristic curves. Acta Paediatr.

[37] Robin X, Turck N, Hainard A, Tiberti N, Lisacek F, Sanchez JC, Müller M (2011). pROC: an open-source package for R and S+ to analyze and compare ROC curves. BMC Bioinform.

[38] Lund C, Brooke-Sumner C, Baingana F, Baron EC, Breuer E, Chandra P, Haushofer J, Herrman H, Jordans M, Kieling C, Medina-Mora ME, Morgan E, Omigbodun O, Tol W, Patel V, Saxena S (2018). Social determinants of mental disorders and the sustainable development goals: a systematic review of reviews. Lancet Psychiatry.

[39] Perkins NJ, Schisterman EF (2006). The inconsistency of “optimal” cut-points using two ROC based criteria. Am J Epidemiol.

[40] Fluss R, Faraggi D, Reiser B (2005). Estimation of the Youden index and its associated cutoff point. Biom J.

[41] Tobia V, Marzocchi GM (2018). The strengths and difficulties questionnaire-parents for Italian school-aged children: psychometric properties and norms. Child Psychiatry Hum Dev.

[42] van de Looij-Jansen PM, Goedhart AW, de Wilde EJ, Treffers PD (2011). Confirmatory factor analysis and factorial invariance analysis of the adolescent self-report strengths and difficulties questionnaire: how important are method effects and minor factors?. Br J Clin Psychol.

