# Supplementary material for: Validation of the parent version of the Strengths and Difficulties Questionnaire (SDQ) to screen mental health problems among school-age children in Mongolia
Source: BMC Psychiatry. 2021 Apr 29;21:218. doi: 10.1186/s12888-021-03218-x (PMC8086060; doi:10.1186/s12888-021-03218-x)
Supplement: Supplementary file 1 — Additional file 1: Distribution of total difficulties score by sex. [file 12888_2021_3218_MOESM1_ESM.pdf]

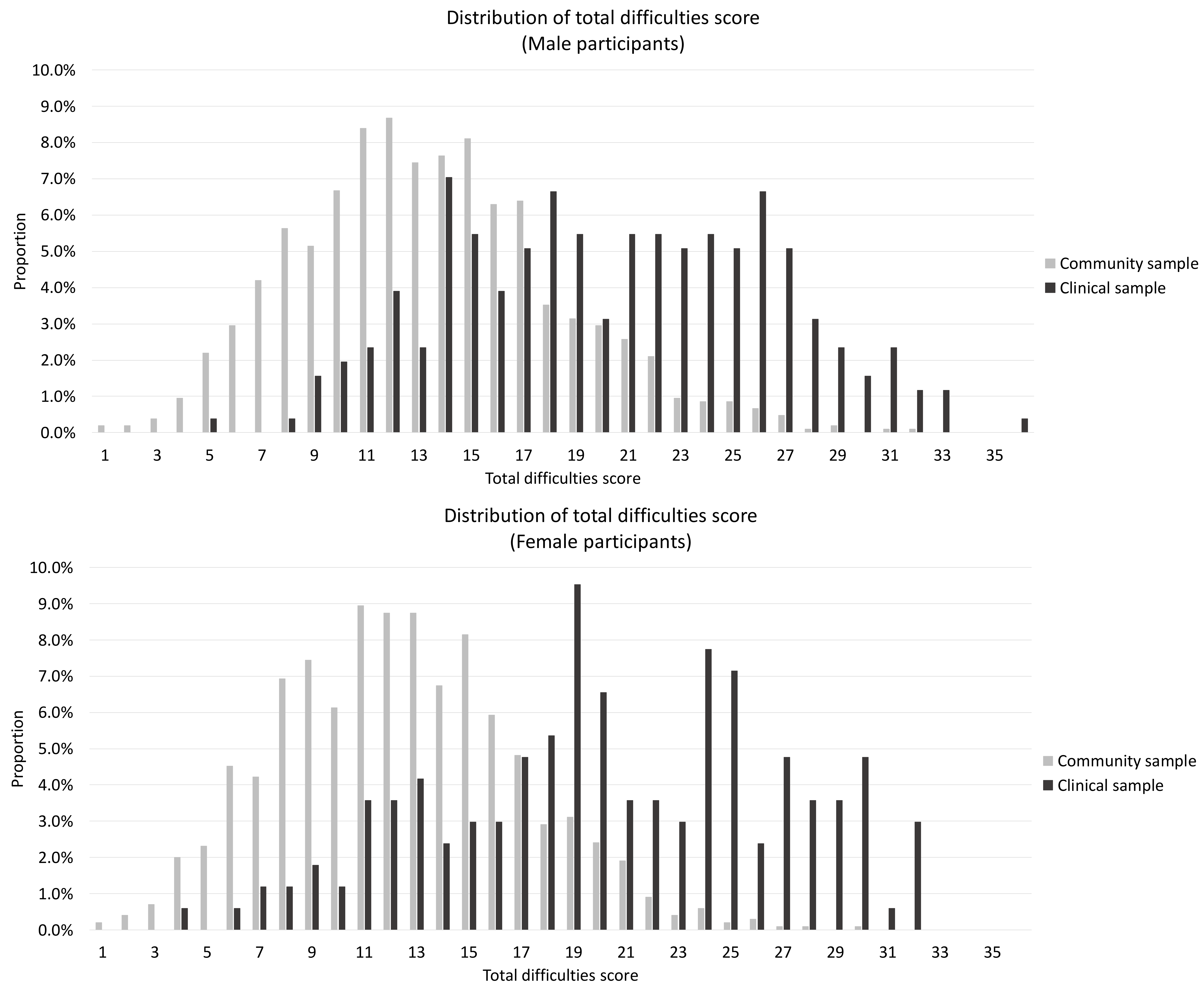
Distribution of total difficulties score
(Male participants)
Proportion
Total difficulties score
Community sample
Clinical sample
Distribution of total difficulties score
(Female participants)
Proportion
Total difficulties score
Community sample
Clinical sample
