# Supplementary material for: Validation of the parent version of the Strengths and Difficulties Questionnaire (SDQ) to screen mental health problems among school-age children in Mongolia
Source: BMC Psychiatry. 2021 Apr 29;21:218. doi: 10.1186/s12888-021-03218-x (PMC8086060; doi:10.1186/s12888-021-03218-x)
Supplement: Supplementary file 2 — Additional file 2: Supplementary Table 1. Normative data of the SDQ total difficulties score. [file 12888_2021_3218_MOESM2_ESM.docx]

Supplementary table 1. Normative data of the SDQ total difficulties score

|  | Total | | Male | | Female | |
| --- | --- | --- | --- | --- | --- | --- |
|  | % | Percentile | % | Percentile | % | Percentile |
| 1 | 0.2% | 0.2% | 0.2% | 0.2% | 0.2% | 0.2% |
| 2 | 0.3% | 0.5% | 0.2% | 0.4% | 0.4% | 0.6% |
| 3 | 0.5% | 1.0% | 0.4% | 0.8% | 0.7% | 1.3% |
| 4 | 1.5% | 2.5% | 1.0% | 1.7% | 2.0% | 3.3% |
| 5 | 2.2% | 4.7% | 2.2% | 3.9% | 2.3% | 5.6% |
| 6 | 3.7% | 8.5% | 3.0% | 6.9% | 4.5% | 10.2% |
| 7 | 4.2% | 12.7% | 4.2% | 11.1% | 4.2% | 14.4% |
| 8 | 6.3% | 19.0% | 5.6% | 16.7% | 6.9% | 21.3% |
| 9 | 6.3% | 25.2% | 5.1% | 21.8% | 7.4% | 28.7% |
| 10 | 6.4% | 31.6% | 6.7% | 28.5% | 6.1% | 34.9% |
| 11 | 8.7% | 40.3% | 8.4% | 36.9% | 8.9% | 43.8% |
| 12 | 8.7% | 49.0% | 8.7% | 45.6% | 8.7% | 52.6% |
| 13 | 8.1% | 57.0% | 7.4% | 53.0% | 8.7% | 61.3% |
| 14 | 7.2% | 64.3% | 7.6% | 60.6% | 6.7% | 68.0% |
| 15 | 8.1% | 72.4% | 8.1% | 68.7% | 8.1% | 76.2% |
| 16 | 6.1% | 78.5% | 6.3% | 75.0% | 5.9% | 82.1% |
| 17 | 5.6% | 84.1% | 6.4% | 81.4% | 4.8% | 86.9% |
| 18 | 3.2% | 87.3% | 3.5% | 84.9% | 2.9% | 89.8% |
| 19 | 3.1% | 90.5% | 3.1% | 88.1% | 3.1% | 93.0% |
| 20 | 2.7% | 93.2% | 3.0% | 91.0% | 2.4% | 95.4% |
| 21 | 2.2% | 95.4% | 2.6% | 93.6% | 1.9% | 97.3% |
| 22 | 1.5% | 96.9% | 2.1% | 95.7% | 0.9% | 98.2% |
| 23 | 0.7% | 97.6% | 1.0% | 96.7% | 0.4% | 98.6% |
| 24 | 0.7% | 98.3% | 0.9% | 97.5% | 0.6% | 99.2% |
| 25 | 0.5% | 98.9% | 0.9% | 98.4% | 0.2% | 99.4% |
| 26 | 0.5% | 99.4% | 0.7% | 99.0% | 0.3% | 99.7% |
| 27 | 0.3% | 99.7% | 0.5% | 99.5% | 0.1% | 99.8% |
| 28 | 0.1% | 99.8% | 0.1% | 99.6% | 0.1% | 99.9% |
| 29 | 0.1% | 99.9% | 0.2% | 99.8% | 0.0% | 99.9% |
| 30-40 | 0.1% | 100.0% | 0.2% | 100.0% | 0.1% | 100.0% |
