# Supplementary material for: Validation of the parent version of the Strengths and Difficulties Questionnaire (SDQ) to screen mental health problems among school-age children in Mongolia
Source: BMC Psychiatry. 2021 Apr 29;21:218. doi: 10.1186/s12888-021-03218-x (PMC8086060; doi:10.1186/s12888-021-03218-x)
Supplement: Supplementary file 3 — Additional file 3: Supplementary Table 2. Normative data of subscales. [file 12888_2021_3218_MOESM3_ESM.docx]

Supplementary table 2. Normative data of subscales

| Score | Emotion subscale | | Conduct subscale | | Hyperactivity/ inattention subscale | | Peer relationship subscale | | Score | Prosocial subscale | |
| --- | --- | --- | --- | --- | --- | --- | --- | --- | --- | --- | --- |
|  | % | pecentile | % | pecentile | % | pecentile | % | pecentile |  | % | pecentile |
| 0 | 4.7 | 4.7 | 16.9 | 16.9 | 3.5 | 3.5 | 5.3 | 5.3 | 10 | 13.6 | 13.6 |
| 1 | 10.2 | 14.9 | 28.9 | 45.8 | 7.0 | 10.5 | 11.7 | 17.0 | 9 | 19.9 | 33.5 |
| 2 | 15.9 | 30.8 | 26.3 | 72.2 | 11.4 | 21.9 | 22.7 | 39.7 | 8 | 20.0 | 53.5 |
| 3 | 18.5 | 49.3 | 15.5 | 87.7 | 14.8 | 36.8 | 24.9 | 64.6 | 7 | 18.3 | 71.7 |
| 4 | 17.2 | 66.5 | 7.6 | 95.3 | 18.2 | 55.0 | 19.1 | 83.7 | 6 | 13.7 | 85.4 |
| 5 | 13.9 | 80.4 | 2.7 | 98.0 | 15.4 | 70.4 | 8.8 | 92.5 | 5 | 8.8 | 94.3 |
| 6 | 9.8 | 90.2 | 1.2 | 99.3 | 10.9 | 81.3 | 5.3 | 97.8 | 4 | 3.4 | 97.7 |
| 7 | 5.3 | 95.5 | 0.5 | 99.8 | 8.3 | 89.6 | 1.7 | 99.5 | 3 | 1.5 | 99.2 |
| 8 | 2.8 | 98.3 | 0.2 | 100.0 | 5.9 | 95.6 | 0.4 | 99.9 | 2 | 0.5 | 99.8 |
| 9 | 1.2 | 99.6 | 0.0 | 100.0 | 2.8 | 98.3 | 0.1 | 100.0 | 1 | 0.2 | 100.0 |
| 10 | 0.4 | 100.0 | 0.0 | 100.0 | 1.7 | 100.0 | 0.0 | 100.0 | 0 | 0.0 | 100.0 |
